# Crystals: animal, vegetable or mineral?

**DOI:** 10.1098/rsfs.2015.0027

**Published:** 2015-08-06

**Authors:** Stephen T. Hyde

**Affiliations:** Department of Applied Mathematics, Research School of Physics and Engineering, The Australian National University, Canberra, Australian Capital Territory 0200, Australia

**Keywords:** crystallography, liquid crystals, form

## Abstract

The morphologies of biological materials, from body shapes to membranes within cells, are typically curvaceous and flexible, in contrast to the angular, facetted shapes of inorganic matter. An alternative dichotomy has it that biomolecules typically assemble into aperiodic structures *in vivo*, in contrast to inorganic crystals. This paper explores the evolution of our understanding of structures across the spectrum of materials, from living to inanimate, driven by those naive beliefs, with particular focus on the development of crystallography in materials science and biology. The idea that there is a clear distinction between these two classes of matter has waxed and waned in popularity through past centuries. Our current understanding, driven largely by detailed exploration of biomolecular structures at the sub-cellular level initiated by Bernal and Astbury in the 1930s, and more recent explorations of sterile soft matter, makes it clear that this is a false dichotomy. For example, liquid crystals and other soft materials are common to both living and inanimate materials. The older picture of disjoint universes of forms is better understood as a continuum of forms, with significant overlap and common features unifying biological and inorganic matter. In addition to the philosophical relevance of this perspective, there are important ramifications for science. For example, the debates surrounding extra-terrestrial life, the oldest terrestrial fossils and consequent dating of the emergence of life on the Earth rests to some degree on prejudices inferred from the supposed dichotomy between life-forms and the rest.

## 1. Introduction

The title of the meeting ‘Bioinspiration of New Technologies' which led to this paper bows to the prevailing *raison d’être* of modern science: in service of modern technology. Surely, the lessons of billions of years of evolution are worth applying to the design and manufacture of new materials and machines. Given my own interest in fundamental research (which after all underpins all game-changing breakthroughs in technology) the following issues came to mind on reflecting on this theme:

(1) What is biology?
(2) How do the physical sciences inform biology?
(3) How does biology inform the physical sciences?
(4) Are the biological and physical universes distinct?

These fundamental questions, which guide the paper, lead to conclusions that—I hope—help to shed some light on how we are to go forward as physical *and* biological scientists.

## 2. Biological form

**Figure 1.**
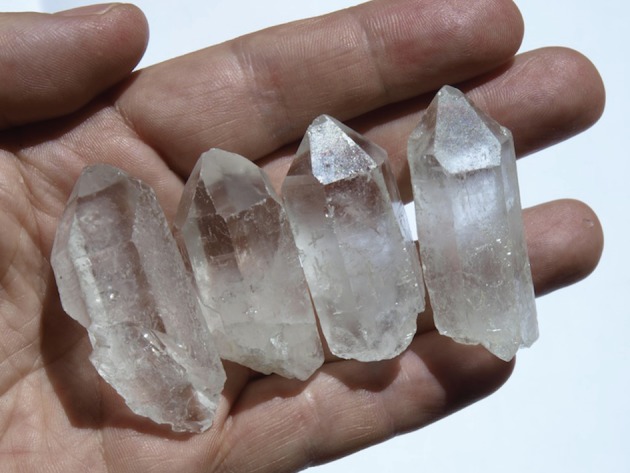
The intuitive view of abiotic or non-living and living materials. Whereas biological forms are curved, inorganic materials are typically facetted and bounded by flat faces. (Image courtesy of JuanManuel Garcia Ruiz).

‘Animal, vegetable or mineral’ was a game we played as children. Someone thought of an object: perhaps a cloud, or a car wheel, or a kangaroo. The object was to identify the object with as few questions as possible, answered by ‘yes' or ‘no’ only. A simple starting question, that narrowed things down pretty rapidly, was to ask ‘Is it animal, vegetable or mineral?’ The game has illustrious antecedents. In the eighteenth century, the pioneering taxonomist, Linnaeus (now known chiefly for his work in plant classification), catalogued the complete spectrum of the material world into three Kingdoms: *Regnum Animale*, *Regnum Vegetabile* and *Regnum Lapideum* [1]. Evidently, animal and vegetables are living, minerals not. And the clues to cataloguing objects into one of those three classes seem clear. For example, living objects, whether animal or vegetable, are typically sinuous, curved and soft; while sterile minerals are typically angular and hard (figure 1).

**Figure 2.**
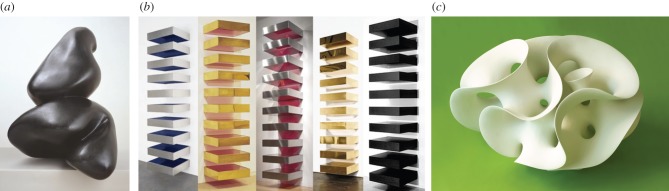
Biomorphic or inorganic sculptural forms? (*a*) Jean Arp, *Fruit du Pagode* (1949); photo copyright Tate, London (2015); (*b*) *Stack* sculptures by Donald Judd (1970s), from [4]; (*c*) Eva Hild, *Funnel Loop 1084* (2007) [2]. (Image courtesy of Eva Hild).

So, for example, ‘biomorphic’ art, design and architecture, developed by the artists Jean Arp, Yves Tanguy and Joan Miró in the last century characterizes living forms as endowed with curvatures, and seemingly more liquid than solid. A beautiful recent example is a sculpture by the Swedish artist Eva Hild [2]. These forms echo the words of the ancient Chinese text *Dao De Jing*: ‘What is supple and yielding goes with life; what is stiff and hard goes with death’ [3]. Examples are shown in figure 2.

A naive answer to the question ‘What is biology?’ emerges from these artistic reflections on biomorphology. Biology is characterized by *curvature*. By contrast, the earliest morphological studies of mineral crystals in the seventeenth century by the Danish founder of crystallography, Steno, recognized that crystals are characterized by fixed angles between *flat* faces. The characteristic flat cleavage faces of crystals were understood by Kepler, Hooke, Huygens and others as arising from regular, ordered arrangements of tiny atoms, much like oranges stacked in a fruit shop.

Those theories were dramatically confirmed with the development of atomic scale crystallography by von Laue and the Braggs, almost exactly 100 years ago. That development arose from the discovery of X-rays, and characteristic diffraction patterns formed by shining X-rays through crystals, first observed by Walter Friedrich and Paul Knipping in 1912. In the hands of the Braggs, and their students, notably William Astbury, Desmond Bernal and Kathleen Lonsdale^1^ crystallography quickly uncovered the atomic arrangements in many inorganic minerals and (later) organic molecular crystals. Crystals are indeed extremely ordered and geometrically rigid stackings of atoms, thereby confirming the earlier ideas of Steno and his successors. While most of Braggs' co-workers continued their explorations of the worlds of mineral and organic crystal, two of their brightest, Astbury and Bernal, decided to explore the biological world via X-ray diffraction.

**Figure 3.**
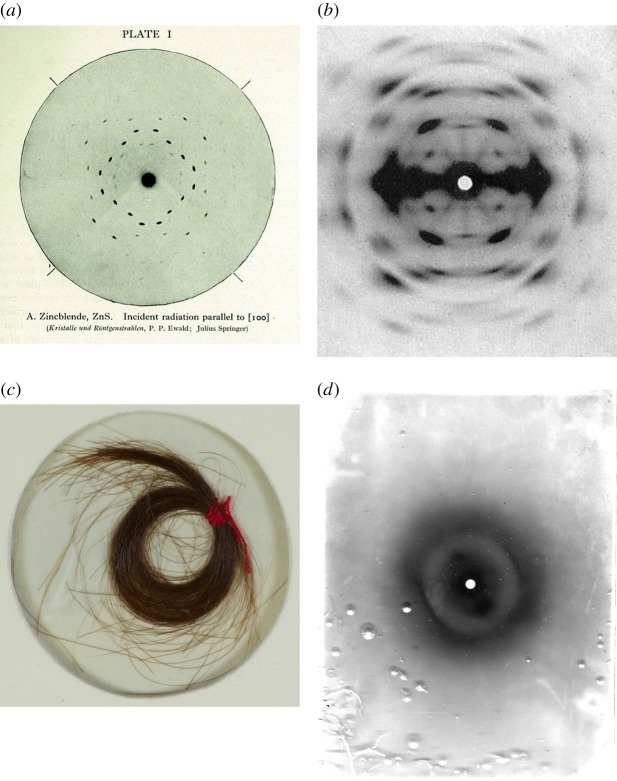
X-ray diffraction patterns from a point source. (*a*) Zincblende (ZnS) crystal diffraction, recorded by P. Ewald; image from *The Crystalline State* by W.H. and W.L. Bragg; (*b*) cellulose fibres, oriented vertically [7]; (*c*) a lock of Mozart's hair, and (*d*) *α*-keratin diffraction pattern, from Mozart's hair (oriented obliquely) by William Astbury's colleague, Elwynn Beighton, in 1958.

Diffraction relies on highly ordered, indeed crystalline (or quasi-crystalline) arrangements of scattering constituents, namely atoms or, via small-angle X-ray diffraction, molecules. (Here I use the term ‘diffraction’ in the conventional sense of wave interference producing discrete diffraction spots, in contrast to ‘scattering’, which gives diffuse intensity distributions in reciprocal space. Given recent developments with very high powered light sources such as X-ray free-electron lasers, this distinction is fading, with the advent of ‘nano diffraction’ techniques [6]). So the question of whether crystallography is helpful in understanding biological structures is worth asking. As the Russian theoretician of crystalline symmetries, Fedorov, said ‘Crystallisation is death’ [3]! In spite of the perceived gulf between animals and minerals, Bernal and Astbury pushed on, and decided to probe proteins, common to all biological species. They split the potentially unending task into two: Astbury headed off to study fibrous proteins in his X-ray apparatus, while Bernal decided to explore globular proteins. Fedorov's dictum seemed to apply: protein diffraction was a messy affair, dominated by diffuse scattering rather than distinct sharp ‘Bragg’ spots. In contrast to the clean, characteristically ‘spotty’ diffraction patterns from highly crystalline minerals, biological matter was revealed to be less ordered, with a virtual continuum from discrete diffractions spots in the former, to diffuse structure in the latter, illustrated by the examples of figure 3.

Astbury was soon investigating porcupine quills, hedgehog spines, merino wool and human hair. Later in his career, his assistant Elwynn Beighton even imaged a lock of Mozart's hair, obtaining an image that moved Astbury—a talented amateur musician—to shed tears during the delivery of at least one lecture.^2^

The intrinsic fuzziness of fibre protein diffraction patterns, such as *α*-keratin, reflects the lack of long-range crystalline order in their structure.^3^ At the atomic scale, crystallographers seemed to have uncovered a natural division between the ordered crystals of the inorganic world and the messier animate cosmos. This finding echoed Fedorov's dictum cited above.

This apparent separation of biological from inanimate matter is complicated by a striking feature of all known life: it is wet. No water; no life. While living ‘extremophiles' have been found in the anaerobic deep sea, at high temperatures and extremes of pH [9], no life is known for thousands of square kilometres in the Atacama Desert in Chile, the driest region on the Earth. Owing to their inevitable water content, most biomaterials are ‘soft materials', defined by the pioneer of soft matter physics, Pierre Gilles de Gennes, as *complex* (containing, for example, many components) and *flexible*. With the notable exception of the skeletal (hard) parts of an organism, living tissue is soft matter, typically composed of variously aggregated biomolecules swimming in a significant volume fraction of water, all held together by weak (and poorly understood) interactions, including the ubiquitous hydrophobic force and electrodynamic interactions, such as dispersion forces [10]. A remarkable feature of many wet biomolecules—observed by Bernal in dispersions of Tobacco Mosaic Virus in water—is their formation of gel-like states, intermediate to liquids and crystals, dubbed ‘liquid crystals'.

As their name implies, liquid crystals combine structural features of both liquids (disorder) and crystals (order); they are intermediate in nature or *mesomorphic* (to use Friedel's name derived from the Greek word for middle [11]). By convention, we distinguish between *thermotropic* and *lyotropic* liquid crystals. Thermotropic examples are formed on heating up a pure compound as an intermediate, and still somewhat ordered stage, of melting. Typical examples are *cholesteric* liquid crystals, characterized by a relative twist along one axis between the orientations of neighbouring molecules. Therefore, the structure is described by the helical (single) twist orientation vector field: if a molecule is located at a some point in the sample, its orientation is likely to be that of the field at that point (or its inverse). So positional order—characteristic of a crystal—is absent, though orientational order exists. The cholesteric phase has a characteristic length scale, namely the pitch of the helical twist orientational field, which is uncorrelated with the molecular dimensions [12]. By contrast, the ‘partial melting’ of lyotropic mesophases is effected by the addition of solvent (rather than heating). Lyotropic mesophases typically form in mixtures of water with detergent (or lipid); these have positional order at mesoscopic length scales, typically tens to hundreds of times the molecular lengths, but are molten at atomic and molecular length-scales. So-called ‘bicontinuous cubic mesophases' are particularly interesting examples of the lyotropic state (that I discuss below).

Both lyotropic and thermotropic liquid crystals are far from rare in biological materials. Examples of cholesteric and bicontinuous cubic phases abound. For example, Yves Bouligand, a biologist with strong interests in liquid crystal physics, established the presence of cholesteric order in the skeletal parts of a variety of organisms [13]. Joseph Needham, a member of the Club for Theoretical Biology (a group—including Bernal—who established the intellectual basis for much of modern biology), said in 1935 ‘[Liquid crystals are] not merely a model for what goes on in the living cell … but a state of organisation actually found in the living cell’ [14]. Indeed, we now know that liquid crystals are so common in biology that Fedorov's pronouncement should perhaps be rephrased to read: ‘*Liquid* crystallisation is life’!.

Bernal and Astbury's visionary project, that kickstarted no less an enterprise than molecular biology, remains as vital today as when it was formulated almost 90 years ago. Indeed, a large fraction of pharmaceutical and medical research—and beam hours of the world's synchrotrons—focuses on the atomic and molecular architectures of biological materials, largely probed by X-ray crystallography. This is an extraordinary testament to the original vision of Bragg's junior colleagues, and to the importance of fundamental physics in driving biological studies. Today, the central repository for protein structure, the Protein Data Bank, hosts almost a hundred thousand protein and nucleic acid structures, 90% of which are reported from X-ray data (and 10% from NMR analyses) [15].

Although impressive in its scope, the project is not the last word in biology. To date, most X-ray structural analyses have required the growth of true protein *crystals*, necessary in order to obtain sharp, mineral-like diffraction patterns. Since biomolecules are essentially ‘dead’ in the crystalline state, these structures do not necessarily reveal their geometric subtleties found in their native, biologically active state *in vivo*. Furthermore, since many (non-structural) proteins are explicitly constructed to avoid aggregation into larger units, they can only be coaxed to crystallize with added molecules, such as detergents, which surely perturb the usual hydrophobic–hydrophilic balance that is so critical to biological activity. These caveats aside, the continuing efforts to deduce the geometry of biomaterials at the atomic and molecular scales, is driven by a simple principle, common across the life and natural sciences: ‘Structure is function’. As Astbury wrote in 1950:

Molecular biology is predominantly three-dimensional and structural - which does not mean, however, that it is merely a refinement of morphology. It must of necessity inquire at the same time into genesis and function. [16, pp. 6–7]

A useful lesson can be gleaned from this brief overview of biological structure. At first glance biological matter is very different in its macroscopic form from hard, angular crystals. However, the very tool developed to probe crystalline structures—crystallography—is now a powerful key to probing structural biology at the molecular scale. Thanks to the discovery of X-rays and the subsequent rapid development of diffraction physics by the Braggs and their team in England, molecular biology was born.

However, as in all victory tales, this triumphal narrative skirts around a less well-understood issue, namely the nature of crystallinity versus liquid crystallinity and the distinction between structural order and disorder.

## 3. Generalized crystallography

The vexed and often abused concept of structural order was analysed explicitly by Bernal. Later in life, inspired by the discovery of the irrational *α*-helix that Pauling deduced from Astbury's fibrous protein data, as well as virus structures then being uncovered by Caspar and Klug, he called for a radical rethink of just what we mean by a ‘crystal’. The complex structures in biological matter had ‘broken formal crystallography, shattered it completely’. He called for a new ‘generalised crystallography’:

We clung to the rules of crystallography which gave us the 230 space groups as long as we could … it needed Pauling to break them down with his irrational α-helix. And so there are no rules, or the old rules are enormously changed. What we have called crystallography is a particular, small branch of crystallography, three-dimensional lattice crystallography. We are seeing now a generalised crystallography … any kind of a repeat organization is a crystal in this general sense. Protein chains are examples of it, so is DNA, and RNA. They have their own inner logic, the same kind of logic but a different chapter of the logic that applies to the three-dimensional regular lattice crystals [17, preface].

This call remains fresh and challenging to this day. Just as physical crystallography spawned molecular biology, so generalized crystallography—that emerged from the complex assemblies found in proteins and viruses—is of increasing urgency and relevance to abiotic matter. The most celebrated example to date is that of quasi-crystals, first reported in metallic alloys, quintessential examples of non-living matter. More recently, however, quasi-crystals have been found in a variety of soft materials, from polymer melts, to colloidal crystals whose chemical compositions resemble the constituents of biological matter [18]. To my knowledge, definitive recognition of quasi-crystals in a living system is unknown. (However, structural studies of plant chloroplasts by Gunning and colleagues uncovered a plethora of forms, some belonging to Bernal's ‘small branch’ of three-dimensional lattice crystallography and others possibly quasi-crystalline or aperiodic. These are discussed further below).

Surely, in time, many new examples of quasi-crystals will be uncovered, possibly also in biology. It is crucial to remind ourselves of Bernal's dictum that emerged from biological studies: that classical crystals are but one realization of patterned structures. Quasi-crystals are just one more species of general crystal. The point is made very clear by Bernal's junior colleague, Alan Mackay, who, following Bernal's thinking, suggested that quasi-crystals may be found in nature, prior to their discovery in alloys by Shechtman [19]; a discovery crowned by the 2011 Nobel Prize for Chemistry. According to Mackay, the report of quasi-crystals is a ‘a kind of legalistic discovery. It's a discovery of a material which breaks the laws that were artificially constructed. They were not laws of nature; they were laws of the human classificatory system.’ [20].

Bernal called for the development of ‘statistical geometry’ to quantify generalized crystallography. It is sobering to realize that only today, 50 years later, is that call being treated seriously, driven in part by the study of granular materials by physicists, as well as mathematical developments in discrete geometry. The exploration of jammed states of these soft materials [21], similar to Bernal's disordered ‘heaps' [22], as well as glass-crystal transitions are now a respectable—indeed fashionable—area of condensed matter science, driven in large part by the massive growth in numerical simulations. Despite that focus, the nature of disordered ‘glassy’ states, and their place within the spectrum of generalized crystallography, remains unclear. For example, Sharon Glotzer has suggested that glasses of rigid polyhedral forms results from a competition between a number of accessible crystalline forms [23]. So are glasses merely wannabe crystals, or, as claimed by Bernal, something quite distinct? We do not yet know.

Bernal's definition of a generalized crystal as a ‘repeat organization’ deserves some reflection. Its superficial informality belies a radical agenda. In particular, he chooses to ignore any specification of dimension, or space. Certainly, given that materials are embedded in three-dimensional Euclidean space, there are some constraints. However, as he makes clear, the repeat organization could refer to any symmetry or quasi-symmetry, not just those discrete isometries of three-dimensional Euclidean space found in the *International Tables for Crystallography* [24]. Clearly, Bernal's agenda was to broaden structural and crystalline concepts to include the very materials his colleagues were exploring so successfully, from proteins to viruses.

Structural studies of viruses revealed regular assemblies of proteins on curved substrates, rather than conventional three-dimensional crystals packed in space. This structural ‘organization’ was therefore very different from that of conventional three-periodic crystals, quasi-crystalline and statistically random arrays in three-dimensional Euclidean space (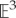). We now know that many viruses are assemblies of capsid proteins on the two-dimensional surface of a (somewhat deformed) sphere (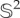). The most symmetric examples result in arrangements with chiral icosahedral point group symmetry (*I* in the nomenclature of the crystallographic community), and form a symmetric decoration of the topological sphere. According to simulations, assembly of two distinct capsomer forms (one viral capsid hexamer and one pentamer), is sufficient to mimic many of the forms observed in viruses [25], though a more basic understanding of just how hexameters and pentamer themselves form is lacking. Nevertheless, the point is clear: icosahedral order emerges from very generic interactions between binary constituent building blocks.

Icosahedral viruses are therefore prime examples of generalized crystals in Bernal's sense. The underlying structural principle is that of a symmetric reticulation (a ‘repeat principle’) of *curved two-dimensional* space (namely 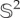) rather than conventional (flat) three-dimensional space. It turns out that symmetric reticulations of another two-dimensional space are equally relevant to materials, whether living or not.

### 3.1. Two-dimensional (non-Euclidean) crystallography

**Figure 4.**
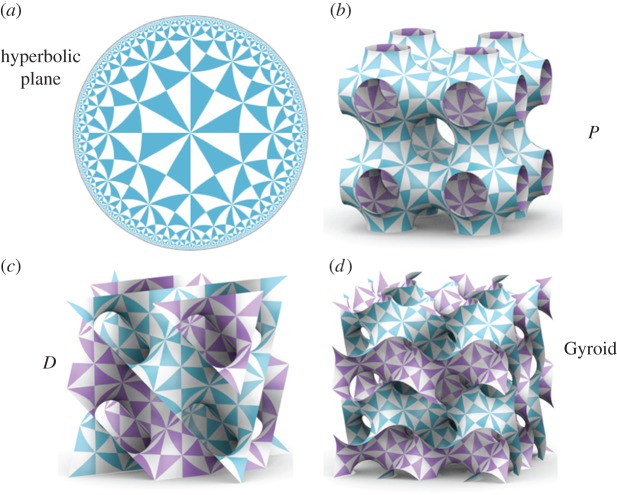
(*a*) Poincaré disc model of the hyperbolic plane (ℍ^2^), tiled with hyperbolic 246 triangles; (*b*) The *P* surface, tiled with 246 triangles; (*c*) The *D* surface, tiled with 246 triangles; (*d*) The Gyroid surface, tiled with 246 triangles. If the blue and white triangles are assumed to be equivalent, all of these patterns display *246 symmetry. (Images courtesy of Myfanwy Evans).

Non-Euclidean geometry admits just three homogeneous two-dimensional spaces, with constant (Gaussian) curvature at all points. Two are well known: the flat plane 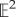 and the sphere, 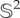. The third space, ‘two-dimensional hyperbolic space’, ℍ^2^, is more difficult to picture, as it is impossible to embed simply in two- or three-dimensional Euclidean space (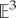). To portray ℍ^2^, some distortions are required. One way, discovered by Poincaré, is to compress it dramatically radially about a single point, so that the entire space sits within a unit disc of 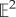. This ‘Poincaré disc model’ of ℍ^2^, has the merit of being conformal, so that all angles in ℍ^2^ are preserved by the map into the flat disc. A pattern in ℍ^2^, drawn in this model, is illustrated in figure 4*a*.

In the past few years, we (and others) have explored repeat organization within ℍ^2^ in some detail. Just as point groups can be derived from isometries of 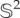, we can enumerate an infinite list of discrete groups that are isometries of ℍ^2^ [26]. While mathematically interesting, it is of little use unless we can map ℍ^2^ into our own space (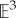). That can be done, with the help of a discrete lattice of disclinations that deform ℍ^2^ just enough to allow it embedding in 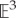 [27]. Within the language of group theory, those embeddings are precisely the subset of the discrete groups of ℍ^2^ that can be mapped into 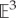 by projection onto hyperbolic surfaces embedded in our space. If we insist that the hyperbolic surface is strictly embedded, i.e. free of self-intersections, the most symmetric examples that we know of are those whose hyperbolic symmetry is described by the Conway orbifold symbol * 246 [26]. Remarkably, it turns out that hyperbolic surfaces with *246 symmetry embed into 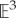 to form multi-handled sponges with three independent lattice vectors, i.e. the surfaces themselves are conventional (cubic) *crystals*. There are three known embeddings, and all three are examples of ‘triply periodic minimal surfaces', namely the *P*, *D* and Gyroid surfaces, illustrated in figure 4.

**Figure 5.**
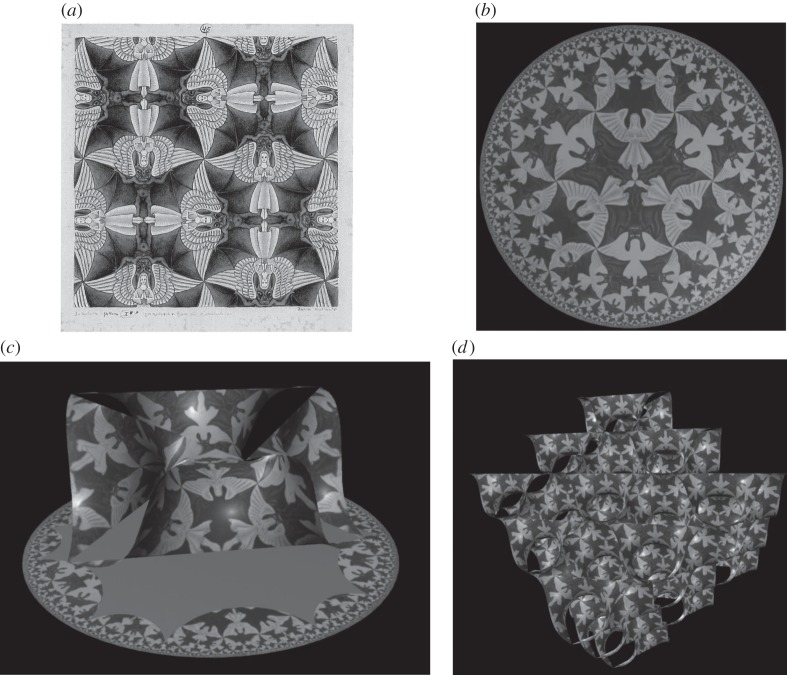
(*a*) M.C. Escher's drawing of a tessellation of the Euclidean plane (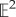), *Angels and Devils* (with orbifold 4*2); (*b*) Escher's hyperbolic *Angels and Devils* tessellation, drawn in the Poincaré disc model of ℍ^2^ (with orbifold 4*3); (*c*) A fragment of the hyperbolic tiling in (*b*), excised from ℍ^2^and mapped into a single (rhombohedral) unit cell of the *P*-surface (a three-periodic minimal surface) embedded in 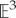; (*d*) A larger fragment of the *P*-surface, made of many unit cells, tiled with *Angels and Devils*. (Images (*b–d*) courtesy of Stuart Ramsden).

We can move between patterns in 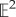 and related patterns in ℍ^2^ with ease, by editing the order of symmetry elements [28]. Consider, for example, a flat two-dimensional crystal, such as the pattern of *Angels and Devils* by Escher, drawn in 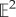. This planar crystalline pattern can be negatively curved, by the addition of disclinations, to form a generalized hyperbolic (two-dimensional) crystal in ℍ^2^, as illustrated in figure 5*a*. Note that in this image the hyperbolic crystal is portrayed within the Poincaé disc model, so that the various angels and devils appear to shrink as they approach the disc edge. In fact, they do not in ℍ^2^, and this effect is an artefact of the map. In the true crystal (i.e. in ℍ^2^), all angels and all devils are identical. The image in figure 5*b* therefore portrays a regular tessellation—and example of Bernal's ‘repeat organization’—of ℍ^2^ with just two tiles, one angelic, the other diabolic. A map of the *Angels and Devils* tiling onto the *P* surface is illustrated in figure 5*c*. Structures that form these surfaces can therefore be viewed through two distinct perspectives. They can be comprehended as patterns embedded within 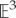, and their triplet of lattice vectors makes them members of Bernal's ‘small branch of crystallography, three-dimensional lattice crystallography’. That view corresponds to the image in figure 5*d*. Alternatively, they can be perceived within the confines of two-dimensional ℍ^2^ alone, rather like the view of a very thin, two-dimensional ant living in the hyperbolic surface. From that perspective, they are two-dimensional, hyperbolically curved crystals, arranged according to the *246 isometries of ℍ^2^. That view corresponds to the tiling of ℍ^2^ itself, shown in figure 5*b*.

The examples in figures 4 and 5 demonstrate a simple principle: regular organizations in ℍ^2^ with suitable symmetries (e.g. *246) can be mapped into conventional three-dimensional crystals in 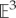. In fact, many three-dimensional Euclidean crystals can be built via this projection from ℍ^2^ to 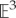. We have enumerated elsewhere the simpler cases of allowed orbifolds [26,29]. Note that most of these *crystallographic hyperbolic orbifolds* project into 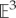 via the *P*, *D* and Gyroid (cubic) surfaces to form crystals that are themselves not cubic, but of lower symmetry. That procedure has been used to generate crystalline three-dimensional nets, via tilings of ℍ^2^ [30–33].

This projection procedure can—in principle—be further generalized to build regular patterns in ℍ^2^ that do *not* project to three-dimensional Euclidean crystals. The situation is somewhat analogous to the formation of irrational helices on a cylinder, formed by rolling up a page decorated with judiciously oriented parallel lines in 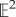. The hyperbolic case is more complex still, since 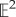 is replaced by ℍ^2^, and the rolling process occurs along a number of axes simultaneously. Although the mathematics has yet to be worked out, and may prove challenging, there is no fundamental obstacle to a fuller enumeration, generating many additional groups whose translational elements are a subset of those of the *P*, *D* and Gyroid surfaces. In the language of crystallography, we could choose translational subgroups of arbitrarily large lattice vectors. Indeed, we could extend one, or two, or all three lattice vectors indefinitely, resulting in a pattern projected onto these surfaces with *no* visible translational symmetries in 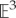
! Such a pattern could remain symmetric in ℍ^2^, with well defined ‘ repeat operations'. In principle then, we can construct generalized crystals (in exactly the sense supposed by Bernal), which contain no translational symmetries. In this manner, hyperbolic space offers immense scope to construct generalized crystals, more exotic than quasi-crystals, or the irrational *α*-helices in proteins.

### 3.2. Minimal surfaces as frustrated mappings from hyperbolic to Euclidean space

In the previous section, the *P*, *D* and Gyroid minimal surfaces have been used as a mathematical scaffold to map from ℍ^2^ to 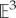. However, it turns out that, in addition to their mathematical utility, these surfaces are routinely realized in condensed materials. These hyperbolic forms are therefore more than geometric abstractions.

Recall that *curvature* is a characteristic of biological form (figure 2). Indeed, the *P* and *D* and Gyroid surfaces and their pairs of intertwined volumes describe well the convoluted labyrinths and hyperbolic lipid-protein bilayers found in many membranes within cell organelles *in vivo*. These are the so-called ‘cubic membranes' [34,35]. The same geometries (albeit shrunken) are also found in abiotic materials that form lyotropic liquid crystals. These are the bicontinuous cubic mesophases. Although these bicontinuous cubic phases are not living materials, their chemical make-up is similar to that of amphiphilic membranes *in vivo*.

The formation of these curved shapes is perhaps unsurprising, given that biomaterials *in vivo* and *in vitro* are typically wet and soft. However, these curved hyperbolic forms were first explored in detail by scientists, due to their relevance to *inorganic* materials. In the early 1980s, Sten Andersson in Lund and Alan Mackay in London suggested independently that the covalent silicate networks of porous zeolites were tilings of the *P*, *D* and Gyroid surfaces [36,37]. So structural curvature, generally thought of as the hallmark of biological form, is prevalent too in inorganic structures. To reiterate, precisely the same structures are formed in living cell membranes, organic lyotropic liquid crystals and inorganic silicates (although at very different lengths scales) [38,39].

Given the very different physics underlying the formation of inorganic silicate structures and soft amphiphilic membranes, the presence of these specific minimal surfaces across such a broad spectrum suggests a fundamental structural feature of these patterns. It is noteworthy that these (and only these) minimal surfaces have *246 symmetry, the smallest orbifold domain of all known hyperbolic orbifolds that can be mapped into 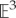. In other words, as far as we know, these surfaces are the most symmetric realizations of homogeneous hyperbolic 2-space in 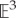. Therefore, the *P*, *D* and Gyroid are likely to represent optimal embeddings of a uniformly curved hyperbolic film [40,41], a hypothesis supported by calculations but as yet unproven [42]. In other words, the fluctuations of (Gaussian) curvature in the *P*, *D* and Gyroid minimal surfaces are smaller than in other more complex minimal surfaces, therefore their bending energy is also lower [40,41,43,44]. If this hypothesis holds true, it implies that the emergence of three-dimensional crystallinity at a global scale in these systems is due solely to a local demand for uniform curvature. Crystallinity then emerges as a frustration-minimizing solution to the incompatibility between ℍ^2^ and 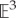.

Despite its importance as a guide to our understanding of the physics of even the simplest hyperbolic structures, this ‘homogeneity’ hypothesis remains unproven. We are hamstrung by mathematical ignorance of alternative candidate structures to these crystalline hyperbolic surfaces. Indeed, we do not even know if quasi-crystalline or aperiodic minimal surfaces of infinite genus exist in 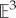, let alone how their curvature homogeneity compares with the simpler cubic *P*, *D* and Gyroid surfaces.

This gap is significant, from the perspectives of both fundamental physics and biology. For, in addition to the *P*, *D* and Gyroid geometries, other, less recognizable hyperbolic structures have also been observed *in vivo* in particularly interesting examples of biological membranes, namely immature and/or light-starved plant chloroplasts. Brian Gunning has reported a number of geometries, all based on hyperbolic surfaces with tetrahedral nodes, forming labyrinths of various forms [45,46]. The structures include conventional crystalline patterns, as well as radially symmetric but aperiodic examples. *None* of these correspond to known minimal surfaces. The existence of these tetrahedral patterns, rather than the better understood *P*, *D* and Gyroid structures, amply exposes our lack of fundamental understanding of two-dimensional hyperbolic patterns in materials, be they biological or non-living. Are other ‘generalized crystals' derived from regular patterns in ℍ^2^, beyond the conventionally crystalline *P*, *D* and Gyroid structures likely to appear in materials? Until we have more fundamental mathematical understanding of hyperbolic surfaces, we do not know. However, it is now certain that regular curved forms are as relevant to our understanding of condensed materials as the familiar facetted forms of hard crystals and curvature is innate in both animate and inanimate materials. At a more philosophical level, once a broader view of just what constitutes a crystal is adopted [47], distinctions between biological and non-living forms at the atomic, molecular and larger length scales are far less clear.

## 4. The organic versus the inorganic

It is helpful at this point to briefly survey other modes of distinguishing the animate and inanimate realms. What, for example, are the *chemical* rather than structural differences between biological and abiotic matter?

Given that most abiotic chemical species can be coaxed to crystallize with relative ease compared with biomolecules, where does the boundary between bio- and abiotic molecules lie? Or, to rephrase the question, can we distinguish *Regnum Lapideum*, *Regnum Vegetabile* and *Regnum Animale* at the atomic or molecular scale? Since the nineteenth century, chemistry has been conventionally divided between ‘organic’ and ‘inorganic’. As the names suggest, the division echoes the animal–mineral dichotomy.

Prior to the twentieth century, organic chemistry was believed to be the study of the fundamental components of living things, those containing a life-force, absent in inorganics. This ‘Vitalism’ philosophy provided a convenient classification principle: in order to belong to *Regnum Animale* or *Regnum Vegetabile*, this mystical life-force was required. The natural corollary was that members of the inanimate kingdom, *Regnum Lapideum* were free of this attribute. To rephrase that dichotomy in the words of perhaps the most celebrated classical physicist and contemporary of Steno, Isaac Newton, ‘Nature's actions are either vegetable … or purely mechanical' [48, p. 306]. It is very curious then to learn that Newton also wrote of the process of ‘vegetation’, that governs ‘Natures obvious laws and processes' and is the ‘sole effect of a latent spirit and that this spirit is the same in all things' [48]. Clearly, Newton is discussing the mystical life-force, since its presence is certainly needed for vegetation. But, he says, it is present in *all* things—animal, vegetable or mineral—writing ‘that metals vegetate after the same laws'. Thus, vitalism permeates all things in Newton's worldview!

That mystical view of Newton's was quickly diluted to a more rational one, in accord with the spirit of the Enlightenment. Until the mid-nineteenth century, the distinction between ‘life’ and the rest was clear: living things were infused with that mystical life-force. However, the rise of organic chemistry in the previous century was driven in part by experiments that proved this simple distinction to be false. Organic molecules could be made in a test tube, from ‘dead’ precursors: the classic example was Wöhler's synthesis of urea from inorganic ammonium cyanate [49] and, in the twentieth century, the Fischer–Tropsch reactions [50]. More recently, geochemists have discovered that complex organic molecules, including polycyclic aromatic hydrocarbons (‘PAHs', until recently, widely assumed to be molecular signatures of life) can be formed under relatively benign hydrothermal conditions, mixing inorganic minerals such as siderite (iron carbonate) and water at elevated temperatures (e.g. [51,52]).

Today, the distinction is reduced to atomic book-keeping only: organic chemistry explores molecules containing carbon atoms, excluding the simple ionic salts (carbonates, oxides and carbides). Inorganic chemistry explores the rest. But in many quarters the prejudice lingers that—somehow—inorganic and organic chemicals are qualitatively different. Given that a typical inorganic crystal (siderite) can be converted to a typical organic molecule (a polyaromatic hydrocarbon) by hydrothermal treatment alone, the old prejudice is surely overdue for disposal.

## 5. Bridging *Regnum Animale*, *Vegetabile* and *Lapideum*

With the benefit of the accumulated knowledge of material structure and function of all species since the seventeenth century, any attempt to unweave the plaited skein that enfolds materials into the three realms proposed by Linnaeus is complex, at best. The discussion in previous sections highlights the pivotal role that liquid crystals play in frustrating a simple classification schema à la Linneaus.

**Figure 6.**
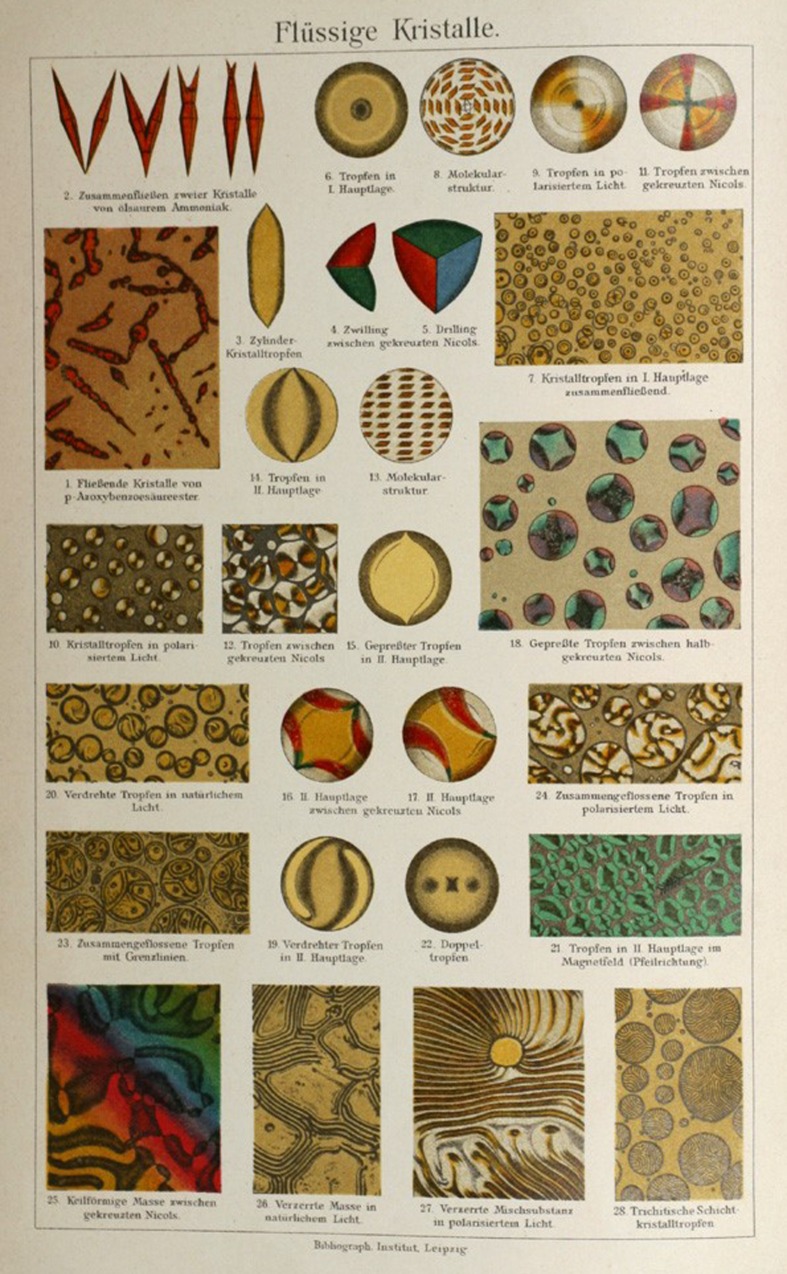
Image from Haeckel's book *Crystal souls* [53].

Liquid crystals were first observed in a class of cholesterol-based organic molecules extracted from plants by the German chemist Reinitzer in the 1880s. With the help of the physicist Lehmann, he had discovered cholesteric liquid crystals. So dramatic and life-like were the writhing figures visible in the optical microscope during the melting process, that the eminent scientist Ernst Haeckel wrote a book entitled ‘Crystal Souls—Studies of Inorganic Life’ (frontispiece reproduced in figure 6) [53]. Haeckel, like Newton before him, guided by his own mystical (and by that time largely outdated) views, was convinced that these liquid crystals contained the essence of life itself, the vital force.

Haeckel's views are generally taken as the last gasp of an outmoded view; the dogma of Vitalism died with him. Ironically, despite the later association of the liquid crystalline state with biological matter by the second half of the twentieth century, the discovery of liquid crystallinity in the nineteenth century triggered much debate, followed by retreat from the dogma of Vitalism. But that retreat was far from orderly, or unanimous. For example, an early twentieth century text described the contributions of Lehmann (the co-discoverer of liquid crystals, thus):

Professor O. Lehmann … endeavoured to prove that no hard and fast line can be drawn between the living and the dead. He contended that crystals of numerous substances showed all the characteristics of life as revealed in certain of the lowest organisms; that substances which crystallise do so in a specific form and resemble many plants; that crystallisation requires a germ to start with; that some crystals are capable of growth, while other poison themselves by absorbing substances contained in the medium investing them. He challenged the statement that living things are always fluid or partially so, and that crystals are invariably solid. In support of this last proposition he maintained that liquid crystals can now be produced … Those of soft soap afford a good example. Professor Lehmann directed attention to some remarkable crystalline forms occurring in viscous fluids which, under the microscope, are seen to be in a state of constant motion. The views of Professor Lehmann have, of course, to be subjected to the most severe criticism on the part of physicists and biologists before they can be accepted … [54, p. 28].

This demise of Vitalism was accompanied by quarantining of physicists from biologists, with occasional—temporary—reunions. Nevertheless, the search for life's specific markers remains. Erwin Schrödinger's essay *What is life?* remains influential today, though most likely more so among biologists rather than physicists. Schrödinger detected two principal features of life: heritability and spontaneous self-assembly. A third characteristic is often invoked today: *emergence* [55], which describes those characteristics of a system that cannot be ascribed to individual constituents, but by their collective activity [56]. The last quality is a challenge to the traditional reductionist mode of scientific explication, and therefore appealing to some (e.g. systems biology [57]) and equally repellant to others [58]. Necessary though these conditions may be for life, all three features are found in abiotic systems as much as living systems. Heritability is well known to crystal growers: seed crystals afford nuclei for crystal reproduction in a test tube. Self-assembly—at equilibrium, or in dissipative systems—is a feature of almost all soft materials, active or passive regardless of their biological content. The liquid crystals described earlier are self-assemblies, and can be formed *in vivo* or in non-living material (though typically the former are far more swollen that the latter). Lastly, emergence is a characteristic of complex systems, such as soft matter, rather than biology *sui generis* [59]. Are there further characteristics of biology that are peculiar to living systems? Some biologists continue to insist there are. For example, the following quote from a physiologist is a scarcely disguised update of Vitalism: … *life has a third secret not mentioned by Schrödinger. The design of living organisms is not determined by physico-chemical laws* [55]. Cleary, the struggle to identify biology as a free-standing science remains a precious one to biologists.

An alternative view is one that views the biological and non-living realms as a continuum, without clear distinction between one and the other. At one extreme, we have the refractory, facetted matter of classical crystals; at the other the curvilinear, articulated blob that is a developing fetus. Between the two extremes lies a multitude of spatial and temporal organizations common to both the living and the sterile. It remains unknown just how broad (gauged by structural and functional measures) that common zone is. The challenge for physical and biological scientists today is to explore that middle zone without prejudice.

While the systems biologists will likely protest, studies of material self-assembly and organization at the mesoscopic length scale (somewhere between atoms, molecules and the cellular scale) offer a useful view of biological and related materials. And that view reveals the importance of Bernal's concept of generalized crystals, particularly liquid crystals, evoked by Haeckel as the seat of the life-force. Certainly, the exploration of liquid crystals in biology—still on-going—has inspired a number of new ideas and open questions in our understanding of the multitude of shapes and assemblies that characterize biological architecture. And much of the intellectual impetus for those newer ideas arose from condensed matter physics and chemistry. The pioneering studies of biological liquid crystals started by Bernal and colleagues in England, were advanced substantially by Vittorio Luzzati [60] and Yves Bouligand [61] in France and Kaare Larsson in Sweden [62]. Their work demonstrated unequivocally the importance of exploring non-living soft materials *in vitro* to understanding biological assemblies *in vivo* and vice versa.

**Figure 7.**
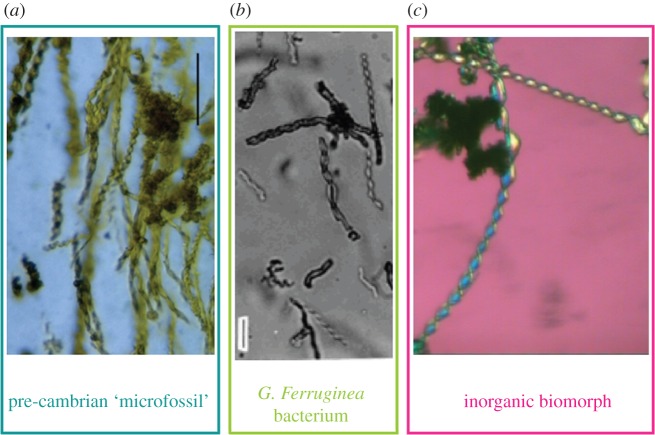
Animal, vegetable or mineral? Three materials, imaged in an optical microscope. (*a*–*c*) An ancient putative micofossil; a bacterium *Gallionella ferruginea* and a silica-carbonate precipitate, respectively.

This lesson—that biology proper (whatever that is) and condensed matter or materials science should be studied hand-in-hand—is one that remains too often ignored. The ‘tyranny of discipline’ remains a major obstacle,^4^ particularly in this era of extreme specialization. One last case study demonstrates the importance—and relevance—of this approach to current science. This example concerns the identification of life's remnants from fossils. Given the mineral composition, induced by templating of earlier biological remnants, the exact location of fossils in Linnaeus' schema is surely problematic. Indeed, structural measures alone are bound to be uncertain. Here is the perfect domain for a challenging game of *Animal, Vegetable or Mineral*! (e.g. figure 7)

**Figure 8.**
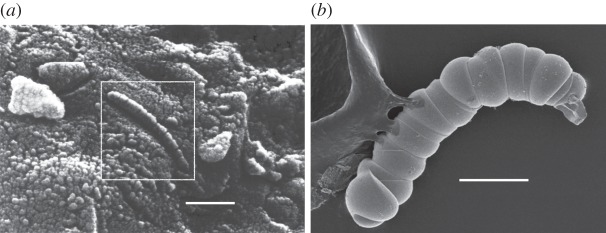
(*a*) NASA's scanning electron micrograph of a fragment of the Martian meteorite, ALH84001, collected in Antarctica. The boxed area shows a segmented structure that resembles a (highly shrunken) bacterium from the meteorite. (Scale bar, 100 µm; estimated from [63]). (*b*) Scanning electron micrographs of silica-barium carbonate ‘biomorphs' grown in the laboratory under sterile conditions. (Scale bar, 30 µm. Image courtesy of Anna Carnerup).

In 1996, NASA triumphantly suggested that they had found the ‘smoking gun’ that demonstrated extra-terrestrial life, namely the presence of bacterial remains within a meteorite dislodged from Mars, and collected in Antarctica [63]. The announcement was met with massive interest, so profound that President Clinton appeared on TV to discuss the finding [64]. The excitement was triggered by a scanning electron micrograph that revealed a curvilinear, segmented shell (figure 8*a*), similar to the shapes of modern filamentous bacteria, and to the forms found in Archaean rocks from northwestern Australia, and believed to be fossilized bacteria.

However, work initiated by Juan Manuel Garcia Ruiz in Granada, has demonstrated that precipitates of carbonate microcrystals in the presence of silica in a test tube at high pH and room temperature also give very similar forms; so life-like that Garcia Ruiz christened them ‘biomorphs' [65]. Subsequent work confirmed that this synthesis readily produces such ‘microfossils', complete with an enclosing membrane reminiscent of aged cell walls, in the absence of any biology (e.g. figure 8*b*) [66]! Given those findings, the claims of biogenicity for the Martian meteorite, and indeed the world's ‘oldest known microfossils', dated to 3.4 Gyr, must remain speculative, at best.

Today, supposed fossilized stromatolites, also from NW Australia, are now trumpeted as the oldest fossils on the Earth [67]. Again, the arguments are complex, strong evidence for their biogenicity is adduced from the regular curvilinear forms of these putative fossils. In my view, the evidence is weak and motivated largely by a prevailing prejudice that curved forms are biological, rather than inanimate. Again, palaeontologists must work hand-in-hand with materials physicists, in order to explore potential abiotic explanations for these forms. For example, recent work demonstrates that extraordinarily ‘life-like’ forms can be generated by complex elastic composites [68–70], and those concerned by understanding the genesis of biomorphologies ignore the findings of physical scientists at their peril.

These examples point to but one conclusion: the supposed disjunction between Animal or Vegetable and Mineral is, on close inspection, fiction rather than fact. We need not return to the extreme viewpoint of Newton, who suggested the ‘vegetation of metals'. However, once we admit the possibility generalized crystals and curvature as a language of forms shared by the animate and inanimate worlds, Linnaeus' rigid classification must be rejected. As Astbury's comment above reminds us, however, biology is function as well as form. The issue of biological function is another chapter, itself worth exploration in detail. Clearly, the extraordinary adaptability and functioning of biological matter far exceeds current biomimetic functioning materials. Here too, however, given the scientific efforts directed in the areas of synthetic biology [71,72] and active matter [73,74], one can predict convergence with some confidence.

The moral of this tale is simply expressed though challenging to implement: biologists, communicate with physical scientists and physical scientists, learn the language of biology!

## Endnotes

1 One of a series of extraordinarily talented and influential women in British crystallography, and later described by Bernal as ‘the presiding genius of the place’ [5].

2 It is indeed a romantic tale, and one may well wonder how a lock of Mozart's hair ended up in Leeds University library! An abbreviated chronology runs thus. The hair was contained within a collection of diaries and papers, presented to Leeds by Donna Nerina Medici di Marignano Gigliucci, a descendent of Vincent Novello (the founder of the British sheet music company, Novello & Co, Ltd). Vincent, who was of Italian origin, visited Italy in 1829 to meet Nannerl Mozart, Wolfgang's elder and (likely) equally talented sister. His diaries of the trip remained in family hands, secreted in Villa Novello, Genoa, after his death. This Villa was occupied by the British Army in the Second World War. The diaries were found by one Major Edward Croft Murray, then quartered in the house, and he passed them on to Donna Medici, who donated them the library.

3 In fact, this lack of crystalline order was already well known to the Braggs. According to Astbury, the first attempt to image X-ray scattering from wool was prepared for his then boss, William Bragg at the Royal Institution, as a Friday evening lecture, entitled *The imperfect crystallisation of common things* [8].

4 An incisive phrase due to Barry Ninham.
